# Anti-NMDA receptor encephalitis unmasking Sjögren’s disease: a case report and literature review

**DOI:** 10.3389/fimmu.2025.1673892

**Published:** 2026-01-13

**Authors:** Gabriel Dimitrov, Vanya Deneva, Stefka Mantarova-Valkova

**Affiliations:** Clinic of Neurology, Military Medical Academy, Sofia, Bulgaria

**Keywords:** anti-NMDA receptor (NMDA-R) encephalitis, autoimmune encephalitis, case report, overlap, Sjögren’s disease, systemic autoimmune disease

## Abstract

Anti-N-methyl-D-aspartate (NMDA) receptor encephalitis is an autoimmune central nervous system (CNS) disorder mediated by antibodies against the GluN1 subunit of the NMDA receptor. Sjögren’s disease (SjD) is a systemic autoimmune disorder that involves exocrine glands as a primary target. However, CNS manifestations, including the coexistence of other CNS diseases, may also occur. While antibodies against the NMDA receptor, targeting the GluN2 subunits, have been associated with SjD and neurological symptoms, the presence of GluN1 antibodies is rarely described, and the co-occurrence of these two disorders has been scarcely reported. Here, we present a case in which anti-NMDA receptor encephalitis and SjD were identified concurrently during the initial workup. The patient experienced three attacks over 13 months, each effectively treated with immunotherapy. No symptoms were reported during the final phone call (month 18). This case report illustrates the ‘unmasking’ of occult systemic autoimmunity by a non-specific neurological syndrome. A comprehensive diagnostic approach is essential to uncover polyautoimmunity and avoid premature diagnostic closure. Further studies may be required to determine whether the association between SjD and NMDA receptor autoimmunity extends beyond the GluN2 subunits to include the pathogenic GluN1 subunit antibodies.

## Introduction

Anti-N-methyl-D-aspartate (NMDA) receptor encephalitis, mediated by immunoglobulin G (IgG) antibodies against the GluN1 (also known as NR1) subunit of the NMDA receptor, is an increasingly recognized autoimmune encephalitis (AE) presenting with neuropsychiatric symptoms, seizures, movement disorders, language, and autonomic dysfunction (1). Sjögren’s disease (SjD) is a chronic systemic autoimmune disorder that primarily targets exocrine glands, but also involves extraglandular manifestations, including the central nervous system (CNS). This can sometimes precede sicca symptoms and co-occur with other autoimmune conditions, such as multiple sclerosis (MS) and neuromyelitis optica spectrum disorder (NMOSD), presenting with multifocal MRI lesions, variable CNS symptoms or asymptomatically (2, 3). The overlap of autoimmune diseases is well recognized, and anti-NMDA receptor antibodies targeting the GluN2 (also known as NR2) subunits have been described in SjD and systemic lupus erythematosus (SLE), where they are associated with cognitive dysfunction and hippocampal atrophy (4, 5). However, the GluN1 (NR1) antibodies, which are pathognomonic for anti-NMDA receptor encephalitis, are only scarcely documented in SjD. Two case reports have described anti-NMDA receptor encephalitis in patients with previously established SjD (6, 7). More recently, a third case report established both diagnoses concurrently on the background of a suggestive clinical picture (8). Here, we present a case in which a non-specific acute neurological presentation led to the concurrent confirmation of definite anti-NMDA receptor encephalitis and SjD.

## Case description

A 39-year-old employed Bulgarian woman presented with a 10-day history of rapid-onset speech disturbances (difficulty articulating words), brain fog, and dizziness. These symptoms emerged shortly after a self-limited fever (subfebrile temperature) and general malaise. Upon further questioning, she reported an isolated psychotic episode following COVID-19 two years ago, for which she was on olanzapine 5 mg, and chronically dry eyes, which she attributed to long screen exposure. No other comorbidities, medications, or unhealthy habits were reported, and there was no family history of genetic disorders. Examination revealed moderate dysarthria, a subtle cognitive deficit (impaired processing/reproduction on testing) with a normal Mini Mental State Examination (MMSE) score, a Glasgow Coma Scale (GCS) score of 15/15, and palpable submandibular glands.

Blood test results, including complete blood count, erythrocyte sedimentation rate, creatinine, urea, electrolytes, aspartate and alanine aminotransferases, C-reactive protein, thyroid-stimulating hormone, creatine phosphokinase, and B12 levels, were unremarkable.

**Figure 1:**
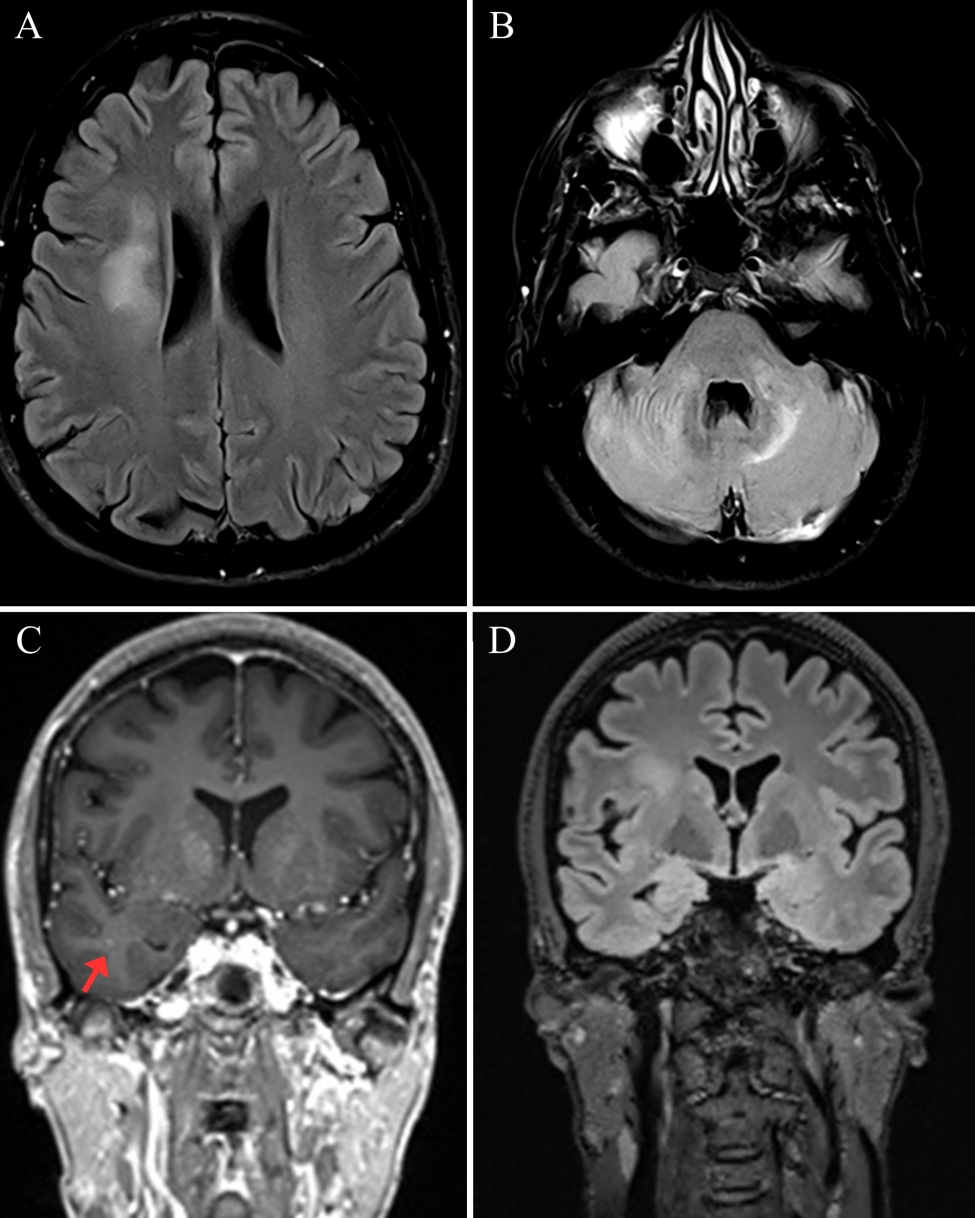
Widespread white matter lesions on brain magnetic resonance imaging (MRI). **(A)** Axial fluid-attenuated inversion recovery (FLAIR) MRI demonstrating an irregularly shaped hyperintense lesion in the periventricular and deep white matter of the right frontal lobe, suggestive of demyelinating or inflammatory etiology. **(B)** Axial FLAIR MRI showing hyperintensities in the cerebellum region, confirming an infratentorial component to the multifocal process. **(C)** Coronal post-contrast T1 at first MRI showing the lack of contrast enhancement in the large right frontal lesion (partially visible) and the presence of a small, avidly enhancing lesion in the right temporal lobe (red arrow), suggesting a focal area of active inflammation. **(D)** A coronal view demonstrating widespread T2/FLAIR hyperintensities involving the periventricular and deep white matter of the cerebral hemispheres, the temporal lobes bilaterally, and a moderate cortical atrophy, a pattern overlapping with encephalitis and various other central nervous system diseases, including manifestations of systemic autoimmunity.

Brain magnetic resonance imaging (MRI) was performed three days before the patient presented at our clinic, revealing multiple bilateral asymmetric T2/Fluid-attenuated inversion recovery (FLAIR) hyperintensities involving the periventricular and deep white matter of the right frontal lobe, temporal lobes, and cerebellum. A small lesion in the right temporal lobe showed contrast enhancement. Moderate cortical atrophy was also observed (**Figures 1A-D**).

Based on these initial findings, the differential diagnosis was wide and included autoimmune or viral encephalitis, acute disseminated encephalomyelitis (ADEM), myelin oligodendrocyte glycoprotein antibody-associated disease (MOGAD), MS, NMOSD, atypical infection, CNS vasculitis, other CNS manifestations of systemic autoimmune disease, and neoplasm. Subsequent diagnostic workup led to two concurrent diagnoses: SjD and anti-NMDA receptor encephalitis (**Supplementary Table 1**).

An electroencephalogram (EEG) was performed and revealed episodes of disorganized slow-wave theta activity during and after photostimulation and hyperventilation. A lumbar puncture was promptly organized. Cerebrospinal fluid (CSF) analysis showed mild lymphocytic pleocytosis (15 cells/μL), elevated protein (0.469 g/L), and CSF-restricted oligoclonal bands (OCBs), indicating intrathecal inflammation. CSF viral polymerase chain reaction (PCR) tests, including herpes simplex virus (HSV), promptly returned negative results. Serum antibody testing for arboviruses also returned negative. Human immunodeficiency virus (HIV), hepatitis B and C, influenza A and B, COVID-19, tuberculosis, syphilis, and Lyme disease were also negative. CSF and serum samples for AE antibody panel were sent to external laboratory.

Concurrently, in the context of her mild sicca symptoms, the diagnosis of SjD was investigated using the 2016 American College of Rheumatology/European League Against Rheumatism (ACR/EULAR) classification criteria (required score ≥4). First, Schirmer test was performed and confirmed bilateral ocular dryness (2 mm/5 min – 1 point). This led to antinuclear antibodies (ANA) screening, which was positive, and subsequently, a separate positive serum anti-SSA/Ro antibody testing, which met the threshold for classification (three points, total four). However, a possible polyautoimmunity could not be excluded at that time.

**Figure 2:**
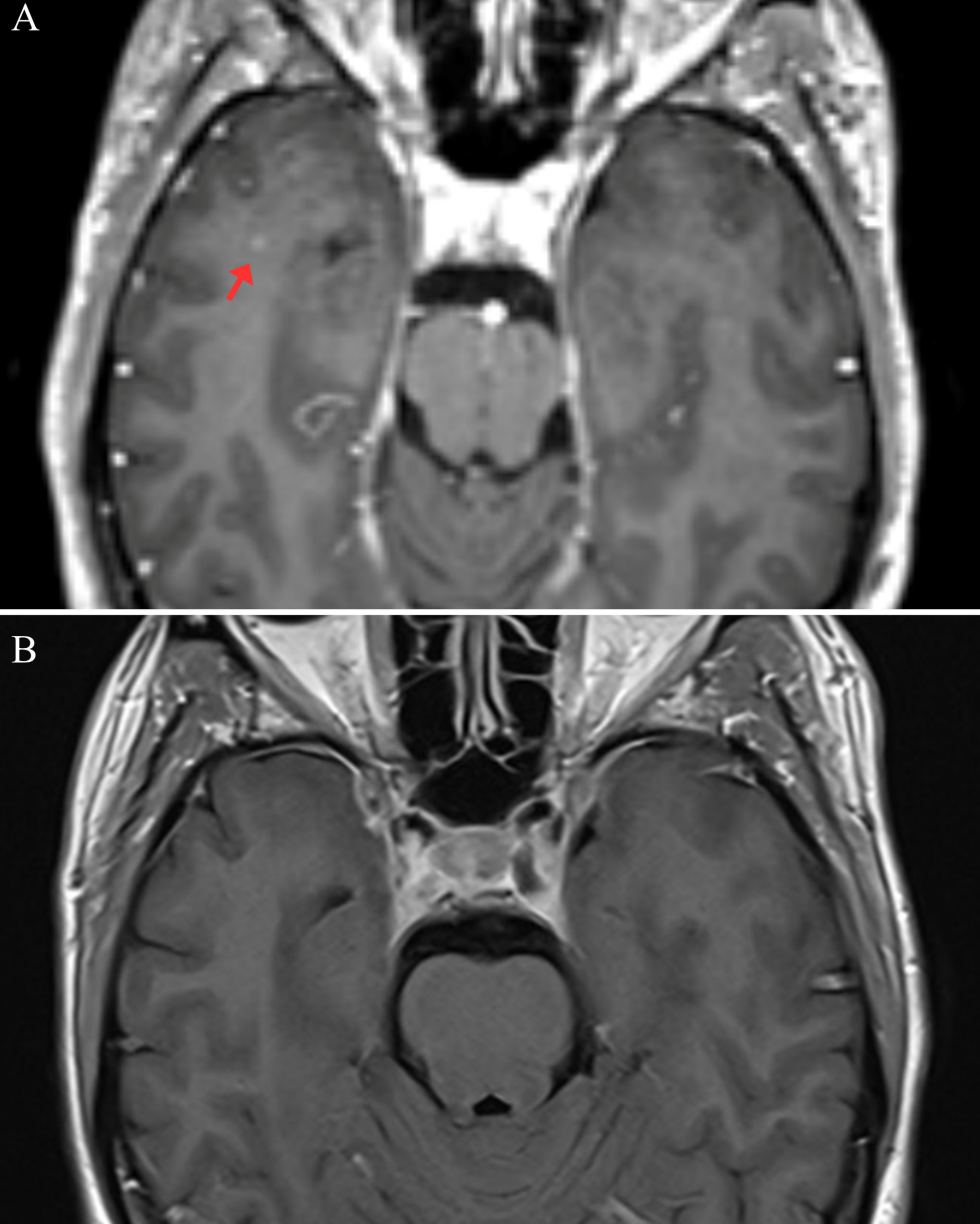
The evolution of contrast-enhancing lesion. **(A)** Axial post-contrast T1 at first magnetic resonance imaging (MRI) clearly shows the small, intensely enhancing lesion in the right temporal lobe (red arrow) prior to treatment. **(B)** Axial post-contrast T1 on the second MRI, day five post-corticosteroid administration, demonstrates a complete resolution of contrast enhancement in the previously enhancing right temporal lesion, which supports a steroid-responsive inflammatory pathology rather than a vascular process.

Given the acute clinical presentation, the presence of CSF pleocytosis and OCBs, and negative viral tests, empirical treatment with high-dose intravenous methylprednisolone (IVMP; 1000 mg daily for 5 days) was initiated promptly while awaiting the results of the AE panel. A second brain MRI on day five of IVMP treatment showed persistence of the previously described T2/FLAIR hyperintense lesions. However, crucially, there was a complete resolution of contrast enhancement in the previously enhanced small right temporal lesion (**Figures 2A, B**). MRI of the cervical spine was normal.

Two weeks post-puncture, the CSF returned positive for anti-GluN1 antibodies (immunofluorescence test, >1:1), and the patient fulfilled the 2016 criteria for definite anti-NMDA receptor encephalitis, while the results of additional serum tests have significantly reduced the likelihood of SLE, antiphospholipid syndrome, rheumatoid arthritis, NMOSD, and MOGAD: normal complement protein levels, absence of SLE-specific antibodies, antiphospholipid antibodies, rheumatoid factor, aquaporin-4 IgG, and myelin oligodendrocyte glycoprotein IgG. Standard exclusion criteria for SjD were also assessed: no history of head or neck radiation, no history or diagnostic suspicion of sarcoidosis, amyloidosis, IgG4-related disease, or graft-versus-host disease.

The patient was discharged, and two months later, she exhibited complete resolution of dysarthria. She underwent abdominal and transvaginal ultrasound examinations and MRI of abdomen and pelvis to screen for ovarian teratomas, all of which were unremarkable. Neurological and rheumatological follow-ups were recommended.

An extended oral prednisone taper over four months was initiated at 60 mg daily, with a dose reduction every two weeks (60 mg → 50 mg → 40 mg → 30 mg → 20 mg → 10 mg → 5 mg → 2.5 mg). However, the clinical course proved to be multiphasic. Seven months after the initial hospitalization (three months following the completion of the first taper), she again presented with mild dysarthria and dizziness, necessitating readmission (first relapse). In a new MRI, the previously large right frontal T2/FLAIR lesion had markedly regressed, while the basal ganglia and cerebellar lesions were described as slightly more prominent. Importantly, no contrast-enhancing lesions were identified. She was treated with a second course of IVMP (1000 mg daily for 5 days). Full recovery was observed during her stay at the clinic, and the same prednisone taper was initiated. However, a second relapse with the same complaints occurred six months after the second hospitalization (two months after the second taper). During this third admission, therapeutic management was escalated to combine immunotherapy with IVMP (1000 mg daily/5 days) and intravenous immunoglobulin (IVIG; 0.4 g/kg/day for 5 days). She was discharged on a rapid prednisone taper (80 mg for 5 days, followed by 2-day steps of 60, 40, 20, 10, 5, and 2.5 mg) and reported resolution of symptoms. Given the relapsing clinical course, the initiation of rituximab was discussed to prevent further recurrence; however, access to this therapy was limited by regional availability and economic constraints. She declined another long-term corticosteroid taper due to significant weight gain. Notably, during this last evaluation, isoelectric focusing revealed no OCBs and repeat serum and CSF panels were negative for anti-GluN1 antibodies. Throughout both relapses, the patient reported no exacerbation of sicca symptoms. Moreover, she suggested improvements but declined further testing. During the most recent phone consultation, conducted four months after the third hospitalization (approximately 18 months following the initial episode), the patient was asymptomatic, remained neurologically stable, and experienced her sicca symptoms so mildly that they were only noticeable upon further questioning.

## Discussion

Simultaneous diagnosis of anti-NMDA receptor encephalitis and SjD during initial diagnostic evaluation of acute neurological syndrome is a notably uncommon and instructive clinical scenario. The literature detailing anti-NMDA receptor encephalitis and SjD is scarce and remains limited to case reports. A comprehensive search of PubMed, Web of Science, and Scopus returned only three recent publications describing patients with SjD developing anti-NMDA receptor encephalitis (6–8). Two cases include patients with already established SjD who subsequently developed anti-NMDA receptor encephalitis (6, 7). However, the development of polyautoimmunity in the presence of an autoimmune condition is a clinically more anticipated scenario. In the elderly case described by Li et al. (6), SjD was already diagnosed 15 years ago, whereas our patient presented with a non-specific neurological syndrome, and the sicca symptoms were undiagnosed and secondary. In the case reported by Çağan et al. (7), SjD was also already established one month prior, and the clinical course was shaped by NMOSD and HSV encephalitis, the latter of which has been well documented and is known to precede anti-NMDA receptor encephalitis, highlighting the expected clinical scenario. Recently, concomitant identification of anti-NMDA receptor encephalitis and SjD during the same presentation has also been reported by Kumar et al. (8) with a more overt neurological syndrome, necessitating appropriate evaluation (**Table 1**). Our case adds to this small body of evidence by illustrating how even ambiguous neurological and sicca symptoms can uncover both anti-NMDA receptor encephalitis and SjD during the initial workup with a structured assessment for coexisting autoimmune diseases and mimics.

**Table 1:** A review of published case reports describing anti-NMDA receptor encephalitis with Sjögren’s disease.

Reference	Patient (age/sex)	SjD status	Neurological context	Anti-NMDA receptor antibody status	Immunotherapy for anti-NMDA receptor encephalitis and clinical outcome
Li et al. (2021) (6)	76-year-old woman	SjD diagnosed 15 years prior (sicca; serology including anti-SSA reported; salivary gland biopsy consistent with lymphocytic sialadenitis)	Progressive cognitive dysfunction with later decreased consciousness and disorientation	Serum anti-NMDA receptor positive (titer 1:10) by CBA	Intravenous methylprednisolone pulse then oral prednisone and mycophenolate mofetil. Marked improvement after treatment; significant improvement reported at 1-year follow-up
Çağan et al. (2022) (7)	52-year-old woman	Diagnosed one month prior to presentation with HSV encephalitis; dry eyes/mouth; anti-Ro/SSA positive; Schirmer positive; salivary gland biopsy grade 4 changes	Severe paraparesis, NMOSD (anti-AQP4 positive) with longitudinally extensive myelitis on MRI, diagnosed simultaneously with SjD and treated with immunosuppression; subsequent HSV encephalitis with altered mental and cognitive status; later post-HSV anti-NMDA receptor encephalitis with persistent symptoms and new MRI changes	CSF NMDA receptor antibody positive at the post-HSV encephalitis phase	Intravenous immunoglobulin (5-day course; continued weekly) then rituximab. Improved orientation and cooperation; residual mild paraparesis noted
Kumar et al. (2025) (8)	“Early 20s”, female	Undiagnosed sicca symptoms for two and a half years; Schirmer positive (3 mm right, 9 mm left); anti-Ro/SSA and anti-La/SSB strongly positive (serum and CSF)	Acute encephalitic syndrome with fever, altered sensorium, focal clonic seizures and dyskinesias; GCS 9/15 at initial presentation	CSF positive on encephalitis mosaic panel (immunofluorescence)	Intravenous immunoglobulin + methylprednisolone with oral steroid taper; antiepileptics; artificial tears; rituximab reported. Improved rapidly; asymptomatic by day 7 and transferred to ward

Anti-NMDA, anti-N-methyl-D-aspartate; AQP4, aquaporin-4; CBA, cell-based assay; CSF, cerebrospinal fluid; GCS, Glasgow Coma Scale; HSV, herpes simplex virus; MRI, magnetic resonance imaging; NMOSD, neuromyelitis optica spectrum disorder; SjD, Sjögren’s disease.

Clinically, distinguishing the primary driver of the neurological syndrome was challenging because both conditions (like many others) can present with neuropsychiatric symptoms, focal neurological deficits, and cognitive dysfunction (2, 9, 10). Our patient did not fulfill the criteria for “probable” anti-NMDA receptor encephalitis at initial presentation, and confirmation relied entirely on the delayed external laboratory analysis. The preceding viral prodrome or infection was suggestive of encephalitis; however, this is also a common feature in ADEM or MOGAD, and infection can trigger flares in NMOSD, MS, and systemic autoimmune diseases (11–13). The history of an isolated psychotic episode following COVID-19 two years ago could possibly represent an unrecognized first episode of anti-NMDA receptor encephalitis or other entity with CNS involvement, but our patient was not evaluated at that time, which underscores the importance of CNS assessment in new-onset psychiatric syndromes. Compared with prior reports, the sicca features in our case were negligible by the patient. The complaint of dry eyes came only after further questioning, and it was attributed to long screen exposure, while the enlarged submandibular glands were only a subjective observation. Ophthalmological consultation with objective eye dryness testing was readily available at our hospital, which led to further serological tests and fulfillment of 2016 ACR/EULAR classification criteria for SjD (14). However, confirming SjD did not exclude the presence of AE, which still awaited confirmation via the antibody testing. During this waiting period, we could not establish whether the condition was caused by a more typical CNS manifestation of SjD or an entirely separate entity, such as AE.

From a neuroimaging perspective, the findings were equally non-specific. The multifocal bilateral T2/FLAIR hyperintensities involving different brain regions were compatible with patterns reported in a similarly wide range of neurological conditions. Medial temporal lobe involvement might favor AE, but is also a common pitfall in misdiagnosis, particularly if combined with extralimbic damage (9). MS-like white matter lesions have been described in systemic autoimmune diseases, but substantial overlap precludes definitive radiological differentiation. The rapid resolution of enhancement in the small temporal lesion post-steroids may suggest an active inflammation responsive to treatment but is non-specific. Attributing lesions to one or another condition was unfeasible.

Given the clinical and radiological ambiguity, the decision to initiate empirical high-dose corticosteroid was made early, following negative viral encephalitis tests. This aligns with recommendations to treat suspected AE empirically (15) and serves to support the claim that this strategy improves outcomes regardless of diagnosis, as other probable entities such as ADEM, MOGAD, MS, and systemic autoimmune disease-associated CNS involvement are also steroid-responsive. On the background of a non-specific neurological syndrome, as in our case, these conditions had to be considered as a possibility while awaiting results. Although ADEM is less common with OCBs (15), it remained a consideration until definitive confirmation of anti-NMDA receptor encephalitis because of the prodromal symptoms. Furthermore, ADEM is a core clinical manifestation of MOGAD as well. CNS demyelinating diseases, including NMOSD and MS, had to be considered, particularly when SjD was established, as it may co-occur with these conditions (2, 3). Excluding the possibility of overlapping connective tissue disorders was also a necessary step.

Beyond these diagnostic and therapeutic considerations, a critical pathophysiological distinction must be drawn between the anti-GluN1 immunity in this case and the anti-GluN2 antibodies previously described in SjD and SLE. Lauvsnes et al. reported that anti-GluN2 antibodies in SjD were associated with worse performance across multiple memory and learning tests and that depression was more frequent among patients with elevated serum anti-NR2 levels, while a subsequent imaging study found lower hippocampal gray matter volume in patients with CSF anti-NR2 positivity (4, 5). The proposed mechanism based on animal models for this phenotype is excitotoxicity: antibodies binding to the GluN2 subunit enhance calcium influx, potentially leading to neuronal apoptosis and irreversible gray matter loss. In contrast, anti-GluN1 antibodies cross-link surface receptors, causing capping or internalization and a titer-dependent, complement-independent, and reversible reduction of synaptic NMDA receptors, producing a state of synaptic hypofunction rather than neuronal loss (1, 16). This could manifest clinically as reversible neurological impairments, as seen in anti-NMDA receptor encephalitis. In an SjD context, anti-GluN1 autoimmunity could plausibly emerge through: (1) B-cell hyperactivity, (2) inflammatory blood-brain barrier (BBB) dysfunction enabling CNS access or intrathecal maturation, and (3) infection-triggered immune activation that primes CNS antigen exposure and intrathecal anti-GluN1 responses. In our patient, a viral infection (if not a prodrome) with the systemic inflammatory setting of SjD may have acted as a permissive environment, disrupting the BBB or enabling intrathecal antibody synthesis. However, clinically, the co-occurrence of these diseases may also simply reflect coincidental comorbidity. A distinct “syndromic overlap” would require evidence of an enriched anti-GluN1 signal in SjD populations or shared immunopathology that is beyond isolated case reports.

Despite reaching definite anti-NMDA receptor encephalitis and SjD diagnoses based on established criteria, the exclusion of other conditions considered in the differential diagnosis remains inherently uncertain, stemming from the potential overlap in clinical and radiological features among these disorders and the limitations of diagnostic tests, including potential false negatives and positives. Therefore, while the presented diagnoses are strongly supported, the possibility of an undetected mimic or genuinely co-occurring pathology cannot be entirely dismissed. The single-case nature of this study further limits its generalizability. Nevertheless, our case report demonstrates that the very need to exclude mimics in the criteria of AE represents a potential for overlooking of other or concurrent pathologies, particularly in the realm of polyautoimmunity. Furthermore, it illustrates the possibility of a delay in the diagnosis of SjD as the course of the disease is insidious, and sicca symptoms are seemingly less important for the patient. What appears to be a “simultaneous diagnosis” reflects a delay in recognizing preexisting SjD rather than a concurrent onset. Systemic manifestations, including neurological ones, are often reported to precede sicca symptoms in SjD (2, 10). However, in this case, chronic dry eyes and subtle gland enlargement were covert and became evident only when the neurological presentation required evaluation. An important unresolved question is whether neurological symptoms genuinely precede sicca symptoms or whether the latter are merely as negligible to the patient, as observed in our case.

In summary, this case contributes to the limited literature on anti-NMDA receptor encephalitis occurring with SjD. Clinicians should maintain a comprehensive approach, particularly when excluding alternative causes in the realm of polyautoimmunity. Whether SjD itself is associated with the production of pathogenic anti-GluN1 antibodies remains to be determined in systematic research.

## Data Availability

The original contributions presented in the study are included in the article/**Supplementary Material**. Further inquiries can be directed to the corresponding author.

## Supplementary material

Supplementary Table 1 Gradual narrowing of the differential diagnosis and criteria fulfillment. * Level of suspicion scale: The numerical scale (1-4) indicates increasing diagnostic certainty based on accumulating evidence: 1 = initial possibility (clinical presentation); 2 = increased possibility; 3 = suggestive objective evidence; 4 = diagnostic criteria met for the respective disease. ACR/EULAR, American College of Rheumatology/European League Against Rheumatism; ADEM, acute disseminated encephalomyelitis; AE, autoimmune encephalitis; ANA, antinuclear antibodies; aPL, antiphospholipid antibodies; APS, antiphospholipid syndrome; AQP4, aquaporin-4; CNS, central nervous system; CSF, cerebrospinal fluid; GVHD, graft-versus-host disease; HIV, human immunodeficiency virus; IgG4-RD, immunoglobulin G4-related disease; JCV - JC Virus; MOG, myelin oligodendrocyte glycoprotein; MOGAD, myelin oligodendrocyte glycoprotein antibody-associated disease; MRI, magnetic resonance imaging; MS, multiple sclerosis; NMDA, N-methyl-D-aspartate; NMOSD, neuromyelitis optica spectrum disorder; OCBs, oligoclonal bands; PCRs, polymerase chain reaction tests; PML, progressive multifocal leukoencephalopathy; RA, rheumatoid arthritis; RF, rheumatoid factor; SAD, systemic autoimmune disease; SjD, Sjögren’s disease; SLE, systemic lupus erythematosus.
