# Supplementary material for: Anti-NMDA receptor encephalitis unmasking Sjögren’s disease: a case report and literature review
Source: Front Immunol. 2026 Jan 13;16:1673892. doi: 10.3389/fimmu.2025.1673892 (PMC12835306; doi:10.3389/fimmu.2025.1673892)
Supplement: Supplementary Table 1 — Gradual narrowing of the differential diagnosis and criteria fulfillment. * Level of suspicion scale: The numerical scale (1-4) indicates increasing diagnostic certainty based on accumulating evidence: 1 = initial possibility (clinical presentation); 2 = increased possibility; 3 = suggestive objective evidence; 4 = diagnostic criteria met for the respective disease. ACR/EULAR, American College of Rheumatology/European League Against Rheumatism; ADEM, acute disseminated encephalomyelitis; AE, autoimmune encephalitis; ANA, antinuclear antibodies; aPL, antiphospholipid antibodies; APS, antiphospholipid syndrome; AQP4, aquaporin-4; CNS, central nervous system; CSF, cerebrospinal fluid; GVHD, graft-versus-host disease; HIV, human immunodeficiency virus; IgG4-RD, immunoglobulin G4-related disease; JCV - JC Virus; MOG, myelin oligodendrocyte glycoprotein; MOGAD, myelin oligodendrocyte glycoprotein antibody-associated disease; MRI, magnetic resonance imaging; MS, multiple sclerosis; NMDA, N-methyl-D-aspartate; NMOSD, neuromyelitis optica spectrum disorder; OCBs, oligoclonal bands; PCRs, polymerase chain reaction tests; PML, progressive multifocal leukoencephalopathy; RA, rheumatoid arthritis; RF, rheumatoid factor; SAD, systemic autoimmune disease; SjD, Sjögren’s disease; SLE, systemic lupus erythematosus. [file Table1.docx]

**Supplementary Table 1. Gradual narrowing of the differential diagnosis and criteria fulfillment**

| **Timeline of evidence** | **Key findings** | **Anti-NMDA receptor encephalitis**  **level of suspicion*** | **Sjögren's Disease**  **level of suspicion** | **Differential diagnosis** |
| --- | --- | --- | --- | --- |
| **Clinical presentation** | Young age, rapid onset, dysarthria, brain fog, dizziness, viral prodrome/infection; history of psychosis; dry eyes; clinically enlarged submandibular glands. | **1** - Clinical phenotype supports encephalitis | **1** - Sicca symptoms & gland enlargement | AE, infectious encephalitis, ADEM, MOGAD, MS, NMOSD, neoplasm, SAD, CNS vasculitis (primary or secondary) |
| **Follow-up observations** | Normal initial blood markers. MRI suggestive of diffuse inflammation. **Ocular Schirmer's test:** 2 mm / 5 min (bilateral). No history or suspicion of head/neck radiation, sarcoidosis, amyloidosis, IgG4-RD, GVHD; **Negative:** CSF viral PCRs; COVID-19, Influenza A and B, Lyme disease, hepatitis B and C, HIV, syphilis, tuberculosis; **CSF analysis:** Lymphocytic pleocytosis (15 cells/μL); Elevated protein (0.469 g/L); CSF-restricted OCBs; **Positive:** ANA screening, Anti-SSA/Ro**.** | **2** - Infectious encephalitis less likely.  **3** - Intrathecal inflammation, supports AE/demyelinating process; OCBs are common in this disease.  Criteria for probable diagnosis not met. | **2** - Key mimics excluded per ACR/EULAR criteria.  **3 -** ACR/EULAR criterions Anti-SSA/Ro (+ 3 points); Schirmer test (+ 1 point)  **4** - ACR/EULAR criteria met (total score 4) | AE, ADEM, MOGAD, MS, NMOSD, SAD, CNS vasculitis (primary or secondary) |
| **Delayed Testing** | **Negative:** RF, SLE-specific antibodies, aPL, anti-AQP4, anti-MOG; Complement C3/C4 normal.  **Positive:** anti-GluN1 antibodies (CSF). | **4** - Meets criteria for definite disease. |  | Both diagnoses confirmed. Excluded or less likely: RA, SLE, APS, MS, MOGAD and NMOSD. |

* Level of suspicion scale: The numerical scale (1-4) indicates increasing diagnostic certainty based on accumulating evidence: 1 = initial possibility (clinical presentation); 2 = increased possibility; 3 = suggestive objective evidence; 4 = diagnostic criteria met for the respective disease. **Abbreviations:** ACR/EULAR, American College of Rheumatology/European League Against Rheumatism; ADEM, acute disseminated encephalomyelitis; AE, autoimmune encephalitis; ANA, antinuclear antibodies; aPL, antiphospholipid antibodies; APS, antiphospholipid syndrome; AQP4, aquaporin-4; CNS, central nervous system; CSF, cerebrospinal fluid; GVHD, graft-versus-host disease; HIV, human immunodeficiency virus; IgG4-RD, immunoglobulin G4-related disease; JCV - JC Virus; MOG, myelin oligodendrocyte glycoprotein; MOGAD, myelin oligodendrocyte glycoprotein antibody-associated disease; MRI, magnetic resonance imaging; MS, multiple sclerosis; NMDA, N-methyl-D-aspartate; NMOSD, neuromyelitis optica spectrum disorder; OCBs, oligoclonal bands; PCRs, polymerase chain reaction tests; PML, progressive multifocal leukoencephalopathy; RA, rheumatoid arthritis; RF, rheumatoid factor; SAD, systemic autoimmune disease; SjD, Sjögren's disease; SLE, systemic lupus erythematosus.
